# Changes to Physical Activity during a Global Pandemic: A Mixed Methods Analysis among a Diverse Population-Based Sample of Emerging Adults in the U.S.

**DOI:** 10.3390/ijerph18073674

**Published:** 2021-04-01

**Authors:** Amanda L. Folk, Brooke E. Wagner, Samantha L. Hahn, Nicole Larson, Daheia J. Barr-Anderson, Dianne Neumark-Sztainer

**Affiliations:** 1 School of Kinesiology, University of Minnesota, Minneapolis, MN 55455, USA; wagn0900@umn.edu (B.E.W.); barra027@umn.edu (D.J.B.-A.); 2 Division of Epidemiology and Community Health, School of Public Health, University of Minnesota, Minneapolis, MN 55455, USA; hahn0203@umn.edu (S.L.H.); larsonn@umn.edu (N.L.); neuma011@umn.edu (D.N.-S.); 3 Department of Psychiatry and Behavioral Sciences, University of Minnesota Medical School, Minneapolis, MN 55455, USA

**Keywords:** COVID-19, physical activity, exercise, young adults

## Abstract

Emerging adults’ lives have changed because of the COVID-19 pandemic. Physical activity (PA) behaviors need to be examined to inform interventions and improve health. Responses to the C-EAT (COVID-19 Eating and Activity over Time) survey (N = 720; age = 24.7 ± 2.0 yrs) were analyzed. This mixed-methods study quantitatively examined changes in self-reported PA (hours/week of mild PA, moderate-to-vigorous PA (MVPA), and total PA) from 2018 to 2020. Qualitative responses on how COVID-19 impacted PA were analyzed using a grounded theory approach. Hours of PA were lower on average for all intensity levels during COVID-19 than in 2018 (p’s < 0.0001). Over half of the sample reported a decrease in MVPA (53.8%) and total PA (55.6%); 42.6% reported a decrease in mild PA. High SES were more likely to report an increase in total PA (*p* = 0.001) compared to those of lower SES. Most (83.6%) participants perceived that COVID-19 had influenced their PA. The most common explanations were decreased gym access, effects on outdoor PA, and increased dependence on at-home PA. Results suggest that emerging adults would benefit from behavioral interventions and health promotion efforts in response to the pandemic, with a focus on activities that can be easily performed in the home or in safe neighborhood spaces.

## 1. Introduction

In early 2020, the World Health Organization declared a global health emergency [1] and the United States announced a public health emergency due to the COVID-19 outbreak [2]. Many aspects of life and health behaviors changed as a result, one of which is physical activity (PA) behavior [3]. A recent review of studies on PA during the COVID-19 pandemic found a consistent decrease in PA across countries, regardless of the type of PA measurement (e.g., min/week or steps/day) [3]. Although average PA has decreased, it is also likely that there is interpersonal variation and that a proportion of the population also saw an increase in PA, possibly due to an increase in time spent at home and increased health concerns. Meeting PA recommendations is a challenge for emerging adults, those at the age bridging adolescence and adulthood (18–29 years), during normal circumstances and a global pandemic undoubtedly introduced additional barriers impacting the amount of PA individuals engage in during a uniquely stressful time.

While it is known that times of stress lead to decreases in PA [4], the current global pandemic offers unique circumstances and barriers that may further impact PA. Beyond the vast physical health benefits of PA [5,6], being active also has numerous mental health benefits, including better stress management, improvement in mood, and reduction in symptoms of depression and anxiety [6]. COVID-19 has increased stress, anxiety, depression, and other poor mental and emotional health outcomes through both illness and quarantine status [7,8,9]. PA may help mitigate the mental and physical outcomes associated with COVID-19. However, societal changes that have occurred due to COVID-19 have likely altered not only the amount of PA people engage in, but also the types, locations, and motivations, which may have long-term implications.

In order to develop effective interventions to reduce the detrimental mental and physical health effects of altered PA, it is imperative to go beyond examining quantitative changes in PA during the pandemic, to explore the complexities of how and why PA has changed. Emerging adults are of particular interest because of their stage of life, often associated with changes in social support, autonomy, and resources [10]. The COVID-19 pandemic has impacted this group by causing uncertainty, insecurity, and instability in multiple areas of daily life [11]. Qualitative research, utilizing behavioral change theory, can help in gaining a deeper understanding of the pandemic’s influences on PA among emerging adult populations. In the current study, the Social Ecological Model is used as a guide to understand how different factors are interconnected to influence and drive complex PA behaviors [12]. Using this model as a framework may be beneficial when examining the impact of COVID-19.

A global pandemic is unprecedented in modern times, but this study aims to give insight as to how to encourage PA engagement during a uniquely stressful time. Understanding emerging adults’ experiences with PA during the COVID-19 pandemic can help inform future interventions and health promotion efforts, particularly because the pandemic is ongoing and effects are likely to continue into a post-pandemic world. Therefore, the purpose of this paper is to use a mixed-methods approach to understand how PA changed during the time of the COVID-19 pandemic in a diverse population-based sample of emerging adults in the United States.

## 2. Materials and Methods

### 2.1. Study Design and Sample

Participants in the C-EAT (COVID-19 Eating and Activity over Time) study were members of the EAT 2010–2018 longitudinal study cohort who were invited to complete an online survey in 2020 during the U.S. outbreak of COVID-19. Participants included young people who attended middle schools and high schools in Minneapolis-St. Paul, Minnesota in 2009–2010 and were followed over time [13,14,15]. The C-EAT survey was designed to capture changes in weight-related health and markers of psychosocial well-being during COVID-19. Email, text message, and mailed invitations were sent during the span of April to October 2020 to the 1568 participants who had completed the most recent follow-up survey in 2017–2018. All participants were mailed a financial incentive following survey completion [9]. Approximately 90% of participants were living in Minnesota at the time of the C-EAT survey. Thus, COVID-19 guidance may have varied from participant to participant. Sociodemographic information for the full sample (N = 720) can be found in Table 1. Participants were emerging adults at the time of the C-EAT survey (mean age = 24.7 ± 2.0 yrs). Based on self-report of gender identity, a majority of the sample were women (62.0%), 36.6% were men, and 1.4% were a different gender.

### 2.2. Survey Measures

The C-EAT and EAT 2018 surveys were based on prior EAT surveys with modifications made to focus recall on weight-related health and markers of psychosocial well-being during the COVID-19 pandemic and prior to the pandemic during the life stage of emerging adulthood [14]. The C-EAT survey measures also included new items designed to assess behaviors relevant to preventing the spread of COVID-19 and how events related to COVID-19 have influenced health behaviors and psychosocial well-being. Survey measures are described below, including the test–retest reliability of EAT 2018 survey measures. Test–retest reliability was assessed using data from 112 participants who completed the survey twice over 3 weeks. Parental socioeconomic status (SES) and ethnicity/race were assessed on the original school-based survey in 2009–2010 [14].

**PA behavior** was assessed using the Godin-Shepherd Questionnaire in both the EAT 2018 and C-EAT surveys [16]. Participants were asked, “In the past week, how many hours did you spend doing the following activities?” with relevant examples for light, moderate, and vigorous intensity. Responses included “None,” “Less than ½ hour a week,” “0.5–2 h a week,” “2.5–4 h a week,” “4.5–6 h a week,” or “6+ hours a week”. The mid-point of each response option was used to calculate weekly hours spent in each intensity (0, 0.3, 1.3, 3.3, 5.3) with the “6+ hours” category coded as 8.0 [17,18]. Intensities were summed to create moderate-to-vigorous PA (MVPA) and total PA variables (Spearman rank-order test–retest *r’s*: mild = 0.55, MVPA = 0.57, and total = 0.55).

**Influence of COVID-19 on PA** was assessed as part of the C-EAT survey only. Participants were asked, “Have recent events related to COVID-19 influenced your physical activity?” Response options were “No,” “Yes, somewhat,” or “Yes, very much.” If either yes option was chosen, participants were prompted with an open-response question, “Please comment on how events related to COVID-19 have influenced your physical activity, including how you get to places (e.g., work, school, the store) and what you do in your free time (e.g., playing sports with friends, exercising at a gym). What event(s) related to COVID-19 have been the most important to your physical activity?”

**Sociodemographic variables** were collected at various timepoints. Structurally racialized categories labeled as race/ethnicity and several indicators of socioeconomic status (SES) were self-reported in the EAT 2010 survey. A three-category SES variable was primarily determined by the highest education level of a participant’s parents and the following additional variables were used to reduce the impact of missing data and to prevent SES misclassification: family eligibility for public assistance, adolescent eligibility for free or reduced-price school lunch, and maternal and paternal employment status [19]. Age in years at each time-point was calculated by subtracting the participant’s birthdate from the C-EAT survey completion date, and gender identity was reported in the C-EAT survey.

### 2.3. Analysis

**Quantitative.** Descriptive statistics were generated for all sociodemographic variables and hours of PA at each intensity. Paired t-test analyses were utilized to compare PA in 2018 to PA during the COVID-19 pandemic. Chi-squares were used to examine differences in percentages of change in PA by sociodemographic groups. All analyses were conducted using SAS Version 9.4 (SAS Institute, Cary, NC, USA).

**Qualitative.** Open-ended, qualitative responses assessing how the COVID-19 pandemic affected PA were analyzed using a grounded theory approach [20]. Codes and themes were not pre-determined but emerged from the data [20,21]. This common methodology for analyzing qualitative data includes describing, organizing, connecting, corroborating, and representing the information gathered from participants. Two independent raters read and coded the data. To create the codebook, the raters worked separately to identify preliminary codes to organize participant responses using a spreadsheet. After codes were finalized, both raters independently coded each comment; codes were not mutually exclusive. Any discrepancy between raters was resolved through consensus. Codes were organized into themes according to levels of the Social Ecological Model [12]. Percentages presented are based on the sample of participants who reported that COVID-19 had affected their PA, regardless of whether they provided additional information for the open-response question (n = 601). All quotes presented have been edited for spelling or grammar corrections with any edits presented in brackets.

## 3. Results

### 3.1. Quantiative Changes in Hours of PA from 2018 to 2020 (during COVID-19)

On average, hours of PA during COVID-19 are lower than during 2018 for mild PA, moderate-to-vigorous PA, and total PA (p’s < 0.0001) (Table 1). Over half of the sample reported a decrease in moderate-to-vigorous PA and total PA (Table 2). There was a statistically significant difference between the proportion of those who reported a decrease, no change, or increase in total PA when comparing SES (*p* = 0.001). Those in the “high” SES category were more likely to report an increase in total PA compared to other SES categories. Other sociodemographic characteristics show similar patterns of change in all intensities of PA with no statistically significant differences (highest proportion reporting a decrease, followed by an increase, followed by no change).

### 3.2. Qualitative Changes in PA Due to COVID-19 Related Events

**Figure 1:**
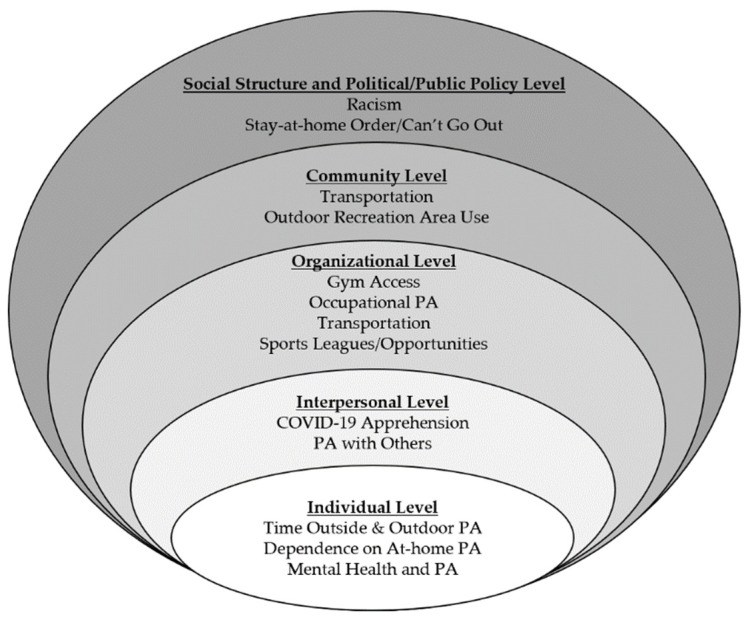
Qualitative Themes Organized based on the Social Ecological Model.

The majority of emerging adults perceived that COVID-19 had influenced their PA either “very much” (n = 312; 43.4%) or “somewhat” (n = 289; 40.2%). Only 118 participants (16.4%) reported no perceived changes in PA due to COVID-19. Themes emerging from the qualitative analysis provide insight as to some of the barriers and facilitators of PA during the COVID-19 pandemic. Using the Social Ecological Model as the framework, twelve themes emerged across the five levels of the model (Figure 1).

#### 3.2.1. Social Structure and Political/Public Policy Level

**Racism.** Although only a small portion of the participants (n = 7; 1.2%) reported racism as something that changed their PA during the COVID-19 pandemic, racism may have an important influence on PA. There are two classes of responses within this theme: those that relate to xenophobia against Asian populations and those who were affected by the uprisings following George Floyd’s murder by Minneapolis police in May 2020 [22].

*“I didn’t feel comfortable going on walks or runs around my neighborhood, especially because I am Asian.”*

*“The recent string of protests and rioting is probably going to impact my physical activity even more.”*

**Stay-at-home Order/Cannot Go Out.** Approximately one fifth of participants (n = 136; 22.6%) specifically cited their state government’s stay-at-home order, guidance to lockdown, or other safety measures to decrease the spread of COVID-19 (e.g., wearing a mask, physical distancing) as a reason their PA has been impacted.

*“The current stay-at-home restrictions have limited the places that I can workout in public.”*

#### 3.2.2. Community Level

**Outdoor Recreation Area Use.** While 9.0% (n = 54) of this sample reported the use of outdoor recreation areas as something that has changed because of the COVID-19 pandemic, a dichotomy emerged in whether they experienced an increase or decrease in the use of outdoor recreation. Some participants indicated that they used outdoor recreation areas more, specifically citing parks, trails, beaches, or lakes; others experienced a barrier to using outdoor recreation areas that they were used to, whether due to outdoor recreation areas being closed or unsafe.

*“My physical activity has decreased since parks, beaches,* etc. *are closed.”*

*“Since the COVID-19 outbreak I do not feel comfortable going outside on trails and paths that I typically frequent.”*

#### 3.2.3. Organizational Level

**Gym Access.** Gym access affecting PA was the second most common theme that emerged from the data. About one third of participants (n = 203; 33.8%) mentioned gyms being closed as a significant barrier to their PA. Some participants mentioned disruption to their preferred routine, such as,

*“Due to all the gyms being closed I have been unable to continue my regular exercise routine.”*

A small portion of the sample (n = 10) mentioned that they would not be returning to the gym regardless of when they open again due to safety concerns or crowding.

**Occupational Activity.** Many respondents had a change in job status because of the COVID-19 pandemic, including loss of employment, a change in shift or numbers of hours worked, or a transfer to working remotely from home. Participants (n = 81; 13.5%) described how these changes led to a change in physical activity. Most of these participants connected it to a decrease in occupational PA and a decrease in overall PA:

*“I get a lot of exercise from work but…I can’t work so I haven’t exercised at all.”*

However, one facilitator of exercise from this consequence of COVID-19 is more free time to be physically active:

*“I am out of work and at home, so I am exercising with my extra time that I have.”*

**Transportation.** A substantial proportion of participants (n = 62; 10.3%) reported that transportation changes affected their PA due to decreased commutes and excursions outside of the home. Most notably, participants reported changes in their active transportation, such as walking, biking, or the use of public transportation:

*“While I’m at school, I walk to get to class…and use public transit and bike shares to get around the city. So not having that regular commute/movement has really reduced my activity.”*

However, others (n = 13) saw active transportation as a new opportunity to be more physically active and introduced active transportation, such as walking to the store instead of driving.

**Sports Leagues/Opportunities.** Participants (n = 42; 7.0%) cited a decreased opportunity for sports as something that affected their PA. They reported cancellations of leagues and classes as well as fewer opportunities for participating in organized sports such as pick-up games, running groups, etc.

*“I would…enjoy soccer/volleyball. But no one wants to risk exposure, so we cancel that now.”*

Participants who reported a decrease in sports mostly cited the cancellation from an organizing body or the social aspect as reasons why these opportunities had decreased.

#### 3.2.4. Interpersonal Level

**COVID-19 Apprehension.** Apprehension around COVID-19 was reported by 9.0% (n = 54) of emerging adults as something that directly affected their PA, particularly out of home PA. One participant reported,

*“The fear of going out and getting sick has kept me from going to exercise…I’m in contact with a lot of people [due to work] so it’s hard to know if I have contact.”*

**PA with Others.** Emerging adults (n = 49; 8.2%) reported that they were counting on roommates, partners, other family members, and/or pets with whom to be physically active. Some noted that they lived with these people, others mentioned meeting up with people outside of their home outdoors.

*“I would also go on a handful of walks during the week with my family or a friend to get out of the house and stay active.”*

*“I go on 3-4 mile runs every morning with my dog and evening walks as well”*.

#### 3.2.5. Individual Level

**Time Outside/Outdoor PA.** The most common theme among respondents (n = 347; 57.7%) was COVID-19 affecting an individual’s choice to be physically active outdoors, though there was a discrepancy concerning how outdoor PA was affected. Just over one fifth (n = 73) of those respondents reported less time outdoors leading to less PA overall. Many participants did note that weather was a factor in their time spent outdoors. The remainder of this subset reported either more time outside being physically active or specifically focusing on outdoor physical activities (e.g., biking, walking, running, hiking). Regardless of the direction, a majority of the respondents made the connection between the ability to choose to be outside and their PA.

*“I think COVID-19 is going to shape how I look at outdoor activity from now on and that it is ALWAYS an option whether it’s in the midst of a pandemic or not.”*

**Dependence on At-Home PA.** Another major theme that emerged (n = 164; 27.3%) was the shift to at-home PA, and the potential challenges of being physically active at home. Just under half of this subset (n = 78) cited an increase in home exercise as compared to before the pandemic, with both aerobic and resistance exercise being reported. Participants also reported making changes to make at-home PA easier, such as purchasing new pieces of equipment and utilizing technology (e.g., Zoom, YouTube) to increase their knowledge of at-home exercise options. However, enjoyment seemed to vary among participants—many indicated not preferring the at-home option.

*“I have tried at-home workouts…but it doesn’t stick as well.”*

**Mental Health and PA.** A portion of the sample (n = 26; 4.3%) reported COVID-19 causing changes in mental health, which then affected their PA. Some of these changes (e.g., increased depressive symptoms) were a barrier to being physically active. However, some participants indicated the importance of PA as a strategy for maintaining their mental health.

*“COVID-19 has caused a bit of depression and lack of energy to get out of bed most days.”*

*“My physical activity changed when I realized how mentally bad I felt after spending an entire day indoors. I have been getting out every day now.”*

## 4. Discussion

The aim of this mixed-methods study was to understand how PA has changed as a result of the COVID-19 pandemic. On average, PA was lower at during the global pandemic than was reported two years earlier for mild PA (−0.52 h/week), moderate-to-vigorous PA (−0.93 h/week), and total PA (−1.47 h/week). A proportion of participants, however, reported an increase of each intensity of PA, suggesting a differential impact of the pandemic on emerging adults, suggesting a need for addressing the needs of those at greatest risk for declines in PA levels. The present study is novel in that it examines how individuals perceive their PA has changed because of the COVID-19 pandemic, providing insight into the barriers and facilitators influencing PA during this global crisis. Results from the study indicate that many aspects of PA have been affected by the pandemic, ranging from alterations in amount, type, and location of PA. Themes emerged at each level of the Social Ecological Model, indicating a complex relationship between the COVID-19 pandemic and PA. Government-mandated lockdowns undoubtedly play a role in PA levels during COVID-19 but are outside of individual control. Systemic racism may also play a role in PA levels and further investigation is needed to explore how best to address. There are three primary areas in which health and fitness professionals may be able to focus their efforts to facilitate increased PA: reduce dependence on gym facilities, encourage outdoor exercise, and facilitate at-home exercise.

The most widely reported barrier to PA during COVID-19 was the closure of gyms and fitness centers. The habit of going to a gym is appreciated by users with diverse PA patterns, with much emphasis on the social support that gyms can provide [23]. As COVID-19 has affected peoples’ schedules and in-person social interaction, it is understandable that the lack of access to a gym would further highlight those changes. Further, individuals who have PA routines are more likely to engage in regular PA [23,24] and the habit of going to a gym likely serves as the routine for these individuals. Therefore, the elimination of their normal routine of going to the gym may have resulted in an overall decrease in PA. As both access to gyms and sport facilities can differ based on socioeconomic status and enjoyment of these facilities is related to athletic abilities and body size, there is likely a health disparity that needs to be addressed based on accessibility to gyms for at risk groups, irrespective of COVID-19 [25,26,27]. However, some individuals in the present study indicated a reliance on a gym or sports facility for PA due to low neighborhood safety or decreased access to public space to be physically active, so gyms should not be discounted entirely. Rather, focusing on using a gym as one of many utilized PA locations, instead of the sole location for PA in health promotion may help create equitable access to PA and assist those who have lost access to gyms to cope with the lack of control over the opening or closing of a fitness center. This mindset may also work to ensure that all people, including those who feel negatively about the gym or cannot access them, can be physically active through future years.

This analysis also highlights the importance of outdoor PA. A substantial proportion of participants reported focusing on their outdoor PA and time spent in natural environments during the pandemic. Thus, outdoor PA is a clear target for health promotion efforts to increase PA at all times, but particularly for coping with effects of the COVID-19 pandemic. There is growing evidence to suggest multiple benefits, both physical and mental, of being physically active outdoors [28,29]. These benefits include decreases in tension, anger, and depression, and an increase in energy levels [28], some which would be of particular importance during pandemic times, due to both the decrease in PA and the increase in stress [9]. Further, because lack of social support for PA and trepidation about COVID-19 were barriers reported by respondents, being active in outdoor settings may help to mitigate lack of social support barriers and simultaneously increase physical activity for subsets of the population if individuals received proper guidance on how they could safely practice physically distanced PA outdoors [30], though more exploration is needed. Adoption of outdoor PA may be weather dependent, which was indicated by some respondents. Participants in the present study indicated that weather is a barrier to outdoor PA, so helping individuals to be prepared and make educated choices about continuing their outdoor PA is imperative for health promotion efforts. Although there are barriers to outdoor PA, it may serve as an important contributor to PA and social support during and after the pandemic.

Finally, there was a shift to at-home exercise reported by the participants, likely due to the systemic changes as a result of the pandemic, leading to more time spent at home and less accessibility to usual PA spaces. There are many activities that lend themselves to at-home exercise including yoga, bodyweight training, active video gaming (exergaming), or aerobic exercise, such as dancing [31]. Additionally, encouraging activities around the house (e.g., cleaning, gardening) can help people achieve the recommended levels of PA, even when they are only in their home environments [32]. Regardless of the plethora of activities, some barriers were identified by the present sample, including limited equipment, limited space to conduct PA, and a decrease in intensity of PA compared to their usual routine. Development of future interventions aiming to increase PA among emerging adults should prioritize home workouts while considering these participant-identified barriers. One mode of at-home exercise that warrants further research is exergaming, which is comparable in intensity to other forms of PA [33,34,35], and is found to be enjoyable by participants [36]. These features of exergaming could increase adherence; exergaming also requires little space, equipment requirements are minimal and certain forms can be free, thereby circumventing many identified barriers to PA.

Study strengths and limitations should be considered when interpreting the findings. To the knowledge of the authors, this is the first qualitative analysis regarding COVID-19 effects on PA in the United States, as well as the first study among emerging adults, a population in which PA has long-term health implications [3]. The present study was able to gain insight from a large, ethnically/racially and socioeconomically diverse population-based sample. Further, because PA was assessed approximately two years before the pandemic, this study allowed for a comparison of PA levels prior to and during the COVID-19 outbreak, allowing us to gain insight into the potential impact of the COVID-19 pandemic on PA. The qualitative methodology uniquely allowed for building understanding of interpersonal variation in how COVID-19 impacted PA. Despite the strengths of the study, it is not without limitations. The survey question regarding how COVID-19 influenced PA gave examples of how PA might have changed (e.g., prompting about transportation or sports, but not about racism), which may have influenced the types of effects reported by participants. Another limitation is the self-reported nature of the assessment of PA, both in 2018 and during the COVID-19 pandemic, as PA is often over-estimated [37]. However, because PA is often over-estimated, it is likely that there is an approximately equal overestimation at both timepoints, which would not skew the changes in time estimations.

There are opportunities to build off the present study that can shape future research and practice. This study did not assess how an individual having COVID-19 themselves or someone in their household having COVID-19 would have impacted PA. Future studies may want to explore how PA has changed for those individuals or families that were directly affected by COVID-19. Additionally, COVID-19 has disproportionately affected people of color in terms of cases, hospitalizations, and deaths [38]. There are disparities in terms of how structural racism and socioeconomic status can affect health behaviors like PA as well [38]. Future studies can focus on how these themes might differ by comparing structurally racialized group experiences and further explore how to minimize PA disparities during a uniquely stressful time.

## 5. Conclusions

The COVID-19 pandemic has brought about a multitude of changes to the lifestyles of many U.S. young people. It is clear that PA has changed in quantity, though not in the same direction for all people. This may lead to larger health disparities post-pandemic that interventions and health promotion programs will need to work to address. It has also changed in terms of what people are doing to be physically active. As society navigates through a pandemic and in a post-pandemic world, it will be imperative to continue monitoring the effects on PA. PA has both physical and mental health benefits that can lessen some of the detrimental effects of a global pandemic. People are adapting their PA in reaction to COVID-19 and the field should look for ways to ensure adaptations that increase PA or make PA the easy option for individuals. Three of those major efforts may be decreasing dependence on gyms, highlighting the importance of outdoor exercise, and helping individuals improve their at-home exercise. Behavioral interventions and health promotion efforts can use information from this study to develop messages tailored to not only what individuals experienced as a result of the pandemic, but who in particular those messages may need to be targeted towards to reduce the impact of the pandemic on future health inequities. PA promotion experts can use this information throughout the remainder of the COVID-19 pandemic, but also for other society-wide challenges in the future.

## Figures and Tables

**Table 1:** Participant Demographics and PA behaviors reported on the C-EAT survey in April–October 2020 (N = 720) ^1^.

Characteristic	Total
Age, *mean ± SD*	24.7 ± 2.0
Gender, *n (%)*	
Female	447 (62.1)
Male	263 (36.5)
Different identity	10 (1.4)
Race/Ethnicity, *n (%)*	
White	213 (29.6)
Black or African American	130 (18.2)
Hispanic or Latino	119 (16.5)
Asian American	172 (23.9)
Native Hawaiian or other Pacific Islander	4 (0.6)
American Indian or Native American	21 (2.9)
Other ^2^	60 (8.3)
Parent Socioeconomic Status, *n (%)*	
Low	231 (32.7)
Middle	279 (37.6)
High	210 (29.7)
Employment Status, *n (%)*	
Working full-time	354 (50.9)
Working part-time	114 (16.4)
Temporarily laid off or unemployed	66 (9.5)
At-home caregiver/not working for pay	162 (23.3)
Living Alone, *n (%)*	
Yes	62 (8.6)
No	658 (91.4)
Living with a parent(s), *n (%)*	
Yes	324 (45.0)
No	396 (55.0)
Living with a child(ren) of your own, *n (%)*	
Yes	122 (16.9)
No	598 (83.1)
Mild Physical Activity (hours/week), *mean ± SD*	
EAT 2018	3.09 ± 2.91
C-EAT	2.57 ± 2.77 *
Moderate-to-Vigorous Physical Activity (hours/week), *mean ± SD*	
EAT 2018	4.55 ± 4.13
C-EAT	3.62 ± 3.84 *
Total Physical Activity (hours/week), *mean ± SD*	
EAT 2018	7.63 ± 6.09
C-EAT	6.16 ± 5.68 *

^1^ Any missing data is due to not responding to the respective survey question. ^2^ “Other” was an option that was self-selected by participants with an optional write-in. * *p* < 0.0001.

**Table 2:** Bivariate associations between changes in PA (% decrease, no change, or increase) from 2018 to 2020 during the COVID-19 pandemic by sociodemographic characteristics.

Characteristic	Mild Intensity PA				Moderate-to-Vigorous PA				Total PA			
	Decrease (%)	No Change (%)	Increase (%)	*p*-Value ^1^	Decrease (%)	No Change (%)	Increase (%)	*p*-Value ^1^	Decrease (%)	No Change (%)	Increase (%)	*p*-Value ^1^
Overall	42.6	26.0	31.4		53.8	11.8	34.4		55.6	6.4	38.1	
Gender ^2^				0.68				0.56				0.90
Female	43.8	26.0	30.2		52.1	11.9	36.0		54.6	6.7	38.7	
Male	40.7	26.2	33.1		55.9	12.2	31.9		56.6	6.1	37.3	
Different identity	40.0	20.0	40.0		70.0	0.0	30.0		70.0	0.0	30.0	
Race/Ethnicity				0.11				0.64				0.16
White	38.5	31.5	30.0		54.5	9.4	36.2		55.4	5.63	39.0	
Black or African American	38.5	26.2	35.4		56.9	12.3	30.8		59.2	7.7	33.1	
Hispanic or Latino	42.0	21.0	37.0		56.3	8.4	35.3		56.3	3.4	40.3	
Asian American	51.7	21.5	26.7		51.7	15.7	32.6		57.0	5.8	37.2	
Native Hawaiian or other Pacific Islander	50.0	0.0	50.0		50.0	0.0	50.0		50.0	0.0	50.0	
American Indian or Native American	52.4	33.3	14.3		52.4	19.0	28.6		66.7	4.8	28.6	
Other	36.7	28.3	35.0		45.0	13.3	41.7		38.3	15.0	46.7	
Parent Socioeconomic Status				0.39				0.21				0.001 *
Low	46.3	23.4	30.3		56.7	12.6	30.7		57.6	6.5	35.9	
Middle	41.9	28.7	29.4		52.7	13.6	33.7		57.4	9.0	33.7	
High	39.5	25.2	35.2		51.9	8.6	39.5		50.1	2.9	46.2	
Employment Status				0.44				0.23				0.28
Working full-time	41.5	24.9	33.6		52.8	10.4	36.7		54.2	5.1	40.7	
Working part-time	45.6	22.8	31.6		58.8	11.4	29.8		54.4	8.8	36.8	
Temporarily laid off or unemployed	53.0	25.8	21.2		62.1	9.1	28.8		66.7	4.6	28.8	
At-home caregiver/not working for pay	39.5	27.8	32.7		49.4	16.7	34.0		53.7	8.6	37.6	
Living Alone				0.90				0.43				0.36
Yes	45.2	24.2	31.5		61.3	11.3	35.1		62.9	3.2	33.9	
No	42.4	26.1	30.6		53.0	11.8	27.4		54.9	6.7	38.4	
Living with a parent(s)				0.22				0.28				0.44
Yes	46.0	25.3	28.7		54.3	13.6	32.1		56.5	7.4	36.1	
No	39.9	26.5	33.6		53.3	10.4	36.4		54.8	5.6	39.6	
Living with a child(ren) of your own				0.52				0.91				0.89
Yes	43.4	22.1	34.4		54.9	12.3	32.8		54.9	7.4	37.7	
No	42.5	26.8	30.8		53.5	11.7	34.8		55.7	6.2	38.1	

^1^ Chi-squares were used to examine differences in percentages of change in PA by sociodemographic groups. ^2^ Chi-square p-values for gender are based on dichotomous categories: non-male vs. male. * *p* < 0.01.

## Data Availability

The data presented in this study are not publicly available but can be provided by senior author Dianne Neumark-Sztainer in response to a reasonable request.
